# A21 MINIMAL MIMETIC MATRICES DRIVE COLONIC ORGANOIDS TOWARD A DISEASE-ASSOCIATED STRESS RESPONSE

**DOI:** 10.1093/jcag/gwae059.021

**Published:** 2025-02-10

**Authors:** V Bat, C Jones, S Nassari, F Boudreau, N Faucheux, N Perreault

**Affiliations:** Universite de Sherbrooke Faculte de Genie, Sherbrooke, QC, Canada; Universite de Sherbrooke Faculte de Medecine et des Sciences de la Sante, Sherbrooke, QC, Canada; Universite de Sherbrooke Faculte de Medecine et des Sciences de la Sante, Sherbrooke, QC, Canada; Universite de Sherbrooke Faculte de Medecine et des Sciences de la Sante, Sherbrooke, QC, Canada; Universite de Sherbrooke Faculte de Genie, Sherbrooke, QC, Canada; Universite de Sherbrooke Faculte de Medecine et des Sciences de la Sante, Sherbrooke, QC, Canada

## Abstract

NOT PUBLISHED AT THE AUTHOR’S REQUEST

Please acknowledge the funding agency below

**Funding Agencies**: CIHR

